# Comparative Analysis of the Effects of Functional Magnetic Stimulation and Interferential Current on Pain Control in Patients With Knee Osteoarthritis

**DOI:** 10.7759/cureus.104073

**Published:** 2026-02-22

**Authors:** Ena Gogić, Edina Tanović, Damir Čelik, Alen Džubur, Nadina Kurtanović, Amela Džubur, Aldijana Kadrić, Dževad Vrabac, Amir Merdović

**Affiliations:** 1 Clinic for Physical Medicine and Rehabilitation, Clinical Center University of Sarajevo, Sarajevo, BIH; 2 PhD Programme in Biomedicine and Health Sciences, Faculty of Medicine, University of Sarajevo, Sarajevo, BIH; 3 Clinic for Heart, Blood Vessels and Rheumatic Disease, Clinical Center University of Sarajevo, Sarajevo, BIH; 4 Department of Physical Medicine and Rehabilitation, Health Institution Spa Gata Bihać, Bihać, BIH; 5 Department of Social Medicine, Faculty of Medicine, University of Sarajevo, Sarajevo, BIH

**Keywords:** functional magnetic stimulation, interferential current therapy, knee osteoarthritis, pain management, physical therapy, visual analog scale

## Abstract

Objective: The objective of this study is to compare the analgesic effects of functional magnetic stimulation (FMS) and interferential current therapy (IFC) in patients with knee osteoarthritis (KOA) before and after treatment.

Methods: This prospective pilot study included 30 patients with KOA, who were randomly assigned to two groups: FMS (n = 15) and IFC (n = 15). Both groups received 20 treatment sessions over four weeks. Pain intensity was assessed using the visual analog scale (VAS) pre- and post-treatment. Non-parametric statistical tests were applied due to the small sample size and non-normal distribution of the data.

Results: In the FMS group, the median VAS score decreased significantly from pre-intervention (Me = 7.0; IQR, 5.0-7.0) to post-intervention (Me = 2.0; IQR, 1.0-2.0), with z = −3.43, p < 0.001. In the IFC group, there was also a significant decrease in the median VAS score from pre-intervention (Me = 7.0; IQR, 5.5-8.0) to post-intervention (Me = 5.0; IQR, 4.0-6.0), z = −3.47, p < 0.001. The Mann-Whitney U test demonstrated a statistically significant difference; the median ΔVAS was significantly higher in the FMS group (Me = 4; IQR, 3.5-6.0) than in the IFC group (Me = 2; IQR, 1.0-2.0) (U = 45, Z = −4.576, p < 0.001).

Conclusion: FMS may be a more effective non-invasive treatment option for pain reduction in patients with KOA compared with IFC.

## Introduction

Knee osteoarthritis (KOA) is one of the most prevalent rheumatic diseases worldwide and a major cause of chronic pain and disability. It most commonly affects knees, hips, the hands (interphalangeal and trapeziometacarpal joints), and the spine, particularly the cervical and lumbar regions. In addition to its clinical impact, KOA imposes a substantial socioeconomic burden through both direct healthcare costs and indirect costs related to reduced productivity and diminished quality of life [1]. The diagnosis of KOA is primarily clinical and is established according to the classification criteria of the American College of Rheumatology (ACR), which incorporate clinical, laboratory, and radiographic parameters. A patient is considered to have KOA in the presence of knee pain accompanied by at least three of the following features: age >50 years, morning stiffness lasting less than 30 minutes, crepitus on active motion, bony tenderness, bony enlargement, absence of palpable synovial warmth, erythrocyte sedimentation rate <40 mm/h, and negative rheumatoid factor. Alternatively, the clinical-radiographic criteria require knee pain in combination with radiographic osteophytes and at least one of the following: age >50 years, morning stiffness <30 minutes, or crepitus on active motion [2]. Radiographic severity is assessed using the Kellgren-Lawrence (KL) grading scale based on standard knee radiographs, which classifies structural changes from grade 0 (no radiographic features) to grade 4 (severe joint space narrowing with large osteophytes, marked sclerosis, and deformity) [3].

KOA represents a significant global public health challenge, highlighting the need for effective prevention and treatment strategies [4]. According to global estimates published in 2021, including data up to 2020, KOA affected 7.6% of the world’s population [5]. The age-standardized prevalence was higher in women than in men, amounting to 8058.9 per 100,000 (95% uncertainty interval (UI) 7251.9-8867.9) for women and 5780.1 per 100,000 (95% UI 5217.8-6341.2) for men. Over the past three decades, the prevalence of KOA has increased by 132.2% and is projected to rise by an additional 60-100% by 2050 [6].

The majority of the disease burden and healthcare costs associated with KOA are attributable to involvement of the knee and hip joints. KOA is now recognized as a chronic inflammatory disease rather than a purely degenerative condition, with pathological processes originating in the synovial membrane and involving activation of the immune system, including humoral and cellular mediators [7].

A variety of physical therapy modalities are used in the management of KOA, including interferential current therapy (IFC), ultrasound, transcutaneous electrical nerve stimulation, laser therapy, kinesitherapy, and hydrotherapy. These modalities demonstrate varying degrees of effect. High-intensity laser therapy achieves the most pronounced reductions in pain intensity measured by the visual analog scale (VAS), whereas ultrasound provides comparatively limited benefit [8].

Several studies have shown that individualized functional magnetic stimulation (FMS) therapy results in significant pain reduction, improved knee joint mobility, and enhanced quality of life, indicating its potential integration into routine clinical practice as a non-invasive treatment option for KOA [9,10]. IFC works by reducing pain, improving circulation, and stimulating muscles [11]. While IFC may offer short- and long-term pain relief and short-term functional improvement, further large-scale, high-quality randomized controlled trials with extended follow-up are required to establish standardized treatment protocols [12].

IFC is widely used in the management of knee osteoarthritis and represents a conventional electrotherapy modality in routine clinical practice. FMS, on the other hand, is a newer, non-contact neuromodulatory intervention that has shown promising analgesic effects in various musculoskeletal conditions. However, despite the growing interest in FMS, direct comparative studies evaluating its analgesic efficacy against established electrotherapy modalities such as IFC remain limited. Therefore, a direct comparison between these two therapeutic approaches is warranted to clarify their relative clinical effectiveness and inform potential evidence-based treatment selection [13-15]. 

VAS is a validated psychometric instrument widely used to assess the intensity of subjective experiences such as pain. It is typically presented as a numerical scale ranging from 0 (no pain) to 10 (worst imaginable pain), allowing patients to quantify perceived pain intensity [16].

The aim of this pilot study was to determine which of the aforementioned therapeutic interventions, including kinesitherapy, more effectively reduces pain intensity in patients with KOA.

## Materials and methods

This prospective comparative study included 30 patients with KOA. The diagnosis of KOA was established based on the clinical presentation and X-ray findings. Radiographic severity was graded using the KL scale. Only patients with KL grade II and III were included in the study. In the IFC group, eight patients had grade II KOA and seven patients had grade III KOA, whereas in the FMS group, six patients had grade II KOA and nine patients had grade III KOA. The duration of KOA is difficult to determine precisely, given that it is a chronic disease. The study was conducted at the Clinic for Physiatry and Rehabilitation, Clinical Center of the University of Sarajevo, between December 2024 and December 2025. Given the absence of prior head-to-head trials comparing FMS and IFC, this study was designed as a pilot investigation to estimate effect size and inform the design of future adequately powered trials. Inclusion criteria were patients aged over 18 years, KOA diagnosed based on clinical examination and radiographic findings, and KOA graded II or III according to the Kellgren-Lawrence classification. Exclusion criteria were surgically treated knee, presence of metal in the knee (due to prior injury, endoprosthesis, or osteosynthetic material), patients with malignancies, presence of a pacemaker, implantable cardioverter-defibrillator, cardiac resynchronization therapy device and pregnancy.

Participants were consecutively recruited and were randomly assigned, using a computer-generated simple randomization list (allocation ratio 1:1), to one of two treatment groups: the FMS group (n = 15) or the IFC group (n = 15). All participants provided written informed consent prior to enrollment.

Both groups underwent a standardized physiotherapy protocol consisting of 20 treatment sessions over a four-week period. FMS therapy was performed using the Tesla Care device manufactured by Iskra Medical (device code: 1800543). The device generates a magnetic field strength of up to 3 T, with a therapy frequency range of 1-160 Hz and four channels. IFC was performed using the Medio MULTI device manufactured by Iskra Medical (device code: 1800241). The device allows a therapy duration of 0 to 99 minutes and features two independent channels. FMS was applied with a magnetic field strength of 1 Tesla at a frequency of 30 Hz, with an active stimulation time of three seconds and a pause of six seconds. We used a medium-sized hand-held applicator, which we positioned over the painful areas of the knee joint for a duration of 15 minutes. IFC was administered at a frequency of 90 Hz using four-pole electrodes. For IFC treatment, four electrodes are used-two placed on the medial side and two on the lateral side of the knee. The currents intersect within the joint, creating interference and stimulating the entire area. The procedure lasted 15 minutes.

In addition to FMS or IFC, all participants received kinesiotherapy. Kinesiotherapy included isometric exercises aimed at strengthening the thigh muscles, performed in three sets of 10 repetitions, with a contraction time of five seconds and rest time of five seconds. During the administration of physical therapy, no patient photography was conducted to ensure the protection of their privacy.

Pain intensity was assessed using the VAS. Measurements were obtained at baseline (pre-treatment) and at the end of the intervention (post-treatment). Statistical analyses were performed using IBM SPSS Statistics for Windows, Version 27 (Released 2019; IBM Corp., Armonk, New York, United States). Continuous variables were presented as medians with interquartile ranges (IQR), and categorical variables as frequencies and percentages. Data normality was evaluated using the Shapiro-Wilk test. Within-group comparisons were conducted using the Wilcoxon signed-rank test, whereas between-group differences were analyzed using the Mann-Whitney U test based on change scores (ΔVAS = VAS_pre − VAS_post). Gender distribution was compared using Fisher’s exact test. A two-tailed p-value <0.05 was considered statistically significant.

Sample size calculation

At the time of study planning, no controlled trials directly comparing FMS and IFC in patients with KOA were available. Therefore, sample size estimation was informed by previous studies investigating magnetic or electromagnetic stimulation for pain reduction in KOA, which have consistently demonstrated large analgesic effects [17,18].

The required sample size was calculated using the following formula:

 $\small n = 2 (Z_{1-\alpha/2} + Z_{1-\beta})^2 / d^2$

where n is the number of participants per group, Z(1−α/2) is the critical value for the significance level α, Z(1−β) is the value corresponding to the statistical power (1−β), and d is the standardized effect size (Cohen’s* d*).

Assuming a large between-group effect size (Cohen’s d ≥ 0.8), a two-group comparison with a two-sided significance level of α = 0.05 and 80% statistical power requires approximately 12-15 participants per group, based on standard sample size estimation formulas for independent group comparisons. Accordingly, a total sample size of 30 participants (15 per group) was considered sufficient for this pilot comparative study, which was primarily designed to estimate treatment effect size and inform future adequately powered randomized trials. 

The Mann-Whitney test provides a robust Z statistic suitable for effect size calculation. Importantly, effect size (r) conveys the magnitude and clinical relevance of the difference between treatments, complementing p-values, which only indicate statistical significance. This approach has been previously described and applied in methodological literature and systematic reviews addressing non-parametric outcomes, supporting its validity and interpretability in studies with small sample sizes. Effect size values were interpreted according to commonly accepted criteria for non-parametric tests: r ≈ 0.10 indicates a small effect, r ≈ 0.30 indicates a moderate effect, and r ≥ 0.50 indicates a large effect. These thresholds are widely applied in rehabilitation, pain research, and clinical outcomes studies. Reporting effect size alongside p-values provides a more comprehensive interpretation of treatment efficacy by quantifying the magnitude of the between-group difference, independent of sample size [19]. 

To compare treatment effects between the two independent groups (FMS vs. IFC), effect size (r) was calculated using the Mann-Whitney test, which is appropriate for non-parametric between-group comparisons:

 $\small r = Z / \sqrt{N}$

where Z represents the standardized test statistic obtained from the Mann-Whitney U test, and N is the total number of observations across both groups. In the present study, 

 $\small N = n_{\mathrm{FMS}} + n_{\mathrm{IFC}} = 15 + 15 = 30$

The absolute value of Z was used to express the magnitude of the effect, regardless of its direction.

Importantly, the reported effect size reflects the magnitude of the between-group difference and complements the p-value by providing information on clinical relevance, rather than merely indicating statistical significance.

## Results

Out of the total sample (n = 30), participants were predominantly women (24 women, 80%) with a smaller proportion of men (six men, 20%). The FMS group consisted of 13 women and two men, whereas the IFC group included 11 women and four men. The difference in sex distribution between the groups was not statistically significant (p > 0.05). The median age in the FMS group was 69 years (IQR, 63.5-75.5), compared with 61 years (IQR, 57.5-71.0) in the IFC group. The age difference between the groups was not statistically significant (p > 0.05). All patients included in the study were in the chronic phase of KOA.

As presented in Table 1, both groups exhibited statistically significant reductions in VAS scores following the intervention.

**Table 1:** Within-group comparison of VAS scores before and after treatment The Wilcoxon signed-rank test was used. IFC: Interferential current therapy; FMS: functional magnetic stimulation; VAS: visual analog scale

Group	Time point	Median (IQR) VAS	z value	p-value
FMS (n=15)	Pre-treatment	7.0 (5.0–7.0)	−3.43	< 0.001
	Post-treatment	2.0 (1.0–2.0)		
IFC (n=15)	Pre-treatment	7.0 (5.5–8.0)	−3.47	< 0.001
	Post-treatment	5.0 (4.0–6.0)		

There was no statistically significant difference in the median baseline VAS score between the FMS (Me = 7; IQR 5.0-7.0) and IFC (Me = 7; IQR 5.5-8.0) groups (U = 92.5, z = −0.855, p = 0.412). In the FMS group, there was a statistically significant reduction in the median VAS score from pre-treatment (Me = 7.0; IQR 5.0-7.0) to post-treatment (Me = 2.0; IQR 1.0-2.0), z = −3.43, p < 0.001. Similarly, the IFC group showed a statistically significant decrease in the median VAS score from pre-treatment (Me = 7.0; IQR 5.5-8.0) to post-treatment (Me = 5.0; IQR 4.0-6.0), z = −3.47, p < 0.001.

As presented in Table 2, the median ΔVAS was significantly greater in the FMS group (Me = 4; IQR, 3.5-6.0) than in the IFC group (Me = 2; IQR, 1.0-2.0) (U = 45, Z = −4.576, p < 0.001).

**Table 2:** Between-group comparison of baseline VAS scores and ΔVAS (Pre-Post) The Mann-Whitney test was used. IFC: Interferential current therapy; FMS: functional magnetic stimulation; VAS: visual analog scale

Outcome	FMS Median (IQR) (n=15)	IFC Median (IQR) (n=15)	z value	p-value
Baseline VAS	7.0 (5.0–7.0)	7.0 (5.5–8.0)	−0.855	0.412
ΔVAS (Pre–Post)	4.0 (3.5–6.0)	2.0 (1.0–2.0)	−4.576	< 0.001

**Figure 1:**
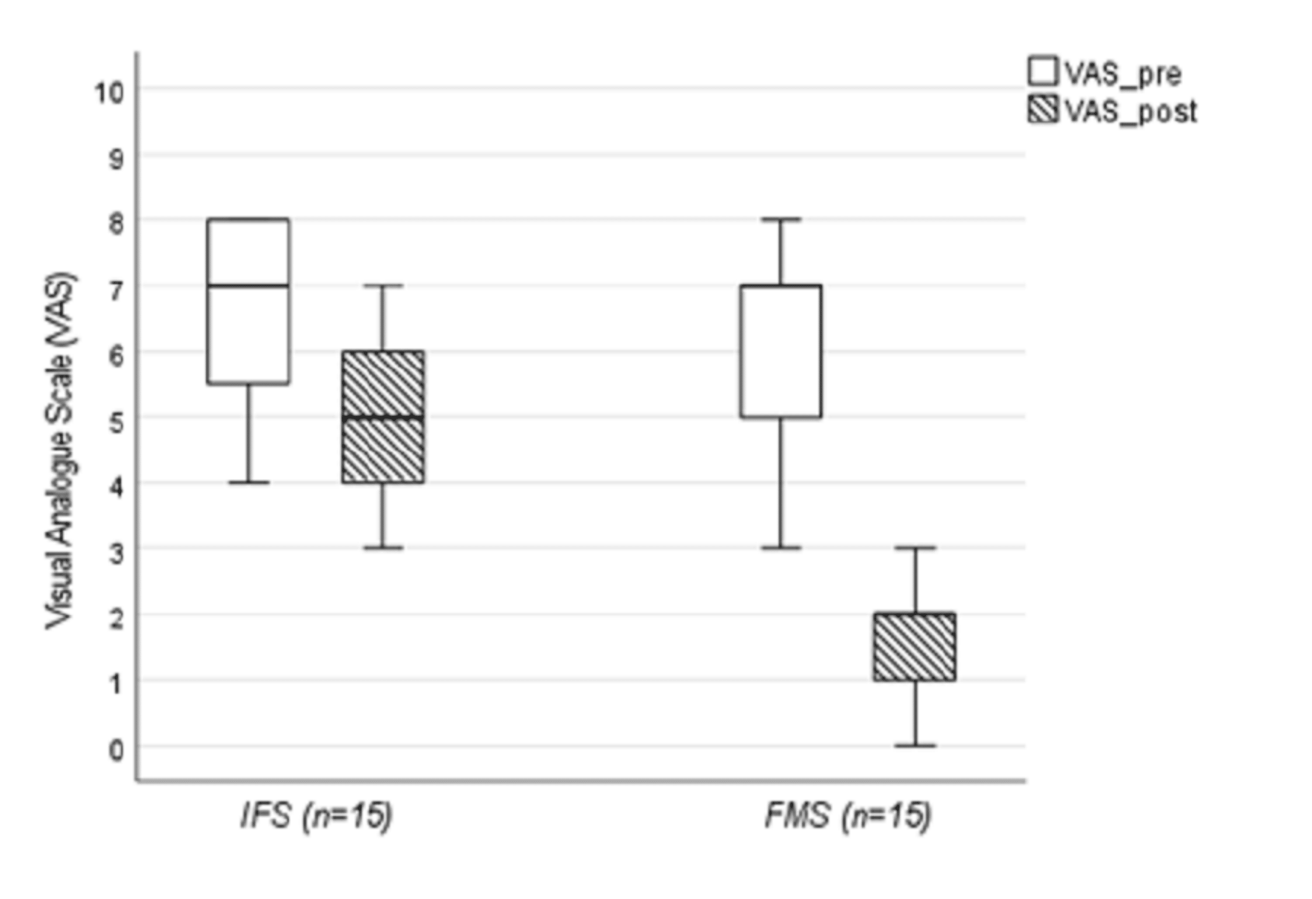
Box plot of the median decrease in VAS scores from pre-intervention to post-intervention for the FMS and IFC groups FMS: Functional magnetic stimulation; IFC: interferential current therapy

As shown in Figure 1, on comparing pain reduction between groups (ΔVAS), the Mann-Whitney test yielded a standardized Z value corresponding to an effect size of approximately r = 0.84.

## Discussion

The present study demonstrated that both FMS and IFC significantly reduced knee pain in patients with KOA; however, pain reduction was significantly greater in the FMS group. These results support the potential clinical application of non-invasive physical modalities in the treatment of KOA.

FMS is a non-invasive modality that delivers time-varying magnetic field pulses generated by electric current to biological tissues [20]. Previous evidence on FMS in KOA is inconsistent. Several randomized controlled trials and systematic reviews have reported contradictory results regarding analgesia and functional improvement. For example, early meta-analytic data suggest that FMS does not consistently reduce pain or improve function compared to placebo, highlighting the need for larger, high-quality studies to confirm its efficacy. Our results, which demonstrate significant pain reduction with FMS, are partially consistent with studies reporting efficacy under certain clinical conditions, but they also highlight variability in the literature, in line with the contradictory outcomes of earlier meta-analyses. On the other hand, other clinical studies have demonstrated favorable effects of FMS on pain and function, particularly when it is applied in combination with physical therapy or when a specific treatment protocol (frequency, duration) is used [21]. 

For example, Hashemi et al. report that the combination of FMS and standard physical therapy leads to significantly greater improvement in VAS and WOMAC functional scores compared to placebo treatment[22]. Electromagnetic fields influence cartilage metabolism, nociception modulation, and inflammatory pathways, although the evidence remains preliminary and depends on treatment parameters. Additionally, comparative studies have reported that FMS provides significantly greater pain reduction and improvement in physical function in early KOA compared to low-level laser therapy [23]. These findings suggest that although electromagnetic therapies may have analgesic and functional effects, the outcomes depend on specific treatment parameters, study design, and patient characteristics. Experimental and clinical evidence suggests that FMS may prevent cartilage degeneration, preserve subchondral bone microarchitecture, and reduce pain through modulation of inflammatory pathways, inhibition of pro-inflammatory cytokines, and stimulation of extracellular matrix production and chondrocyte proliferation [24].

IFC involves the application of medium-frequency alternating currents that intersect within the tissues to produce a low-frequency therapeutic effect. Studies have shown that chronic pain in KOA is associated with central sensitization, and nerve electrical stimulation interventions such as IFC can target central sensitization by modulating pain and desensitizing the central nervous system, resulting in a long-term analgesic effect and, consequently, improved function in patients with KOA. Regarding IFC, systematic reviews and randomized studies generally support its effectiveness in reducing pain and improving functional outcomes in KOA. Chen et al. report that IFC significantly improves short- and long-term pain scores, as well as short-term functional outcomes according to the WOMAC, compared with the control group [11]. Furthermore, Ata et al. also confirm that the application of IFC significantly improves pain intensity and physical function in patients with KOA [12]. These data support our findings that IFC contributes to pain reduction and functional improvement in KOA; however, the effect may be less pronounced compared to FMS, which is consistent with our comparative results.

Differences in the availability and costs of FMS and IFC devices have been described in previous literature, which notes that IFC devices are widely available and financially accessible, whereas FMS systems require greater investment in equipment and staff training [25]. 

These mechanisms help to better understand the clinically observed analgesia of the modality. However, heterogeneity in treatment protocols (frequency, duration, intensity) likely contributes to variability in results, highlighting the need for standardized therapeutic protocols in future research.

Strong evidence supports the short-term benefits of exercise therapy on pain and function in KOA, regardless of exercise type, while therapist supervision may enhance treatment effects [26]. In addition, several adjunctive physiotherapy techniques, including Mulligan mobilization, Pilates, kinesiotaping, and aquatic therapy, have demonstrated effectiveness when combined with conventional treatment approaches [27].

The choice of a 20-session treatment protocol is based on clinical practice and available studies examining exercise programs and electro-physical therapy modalities in KOA. For example, randomized controlled trials in patients with KOA have utilized structured exercise programs combined with modalities over approximately 20 sessions to assess clinical outcomes, providing a basis for the selection of this protocol [28]. Furthermore, systematic reviews and meta-analyses of physical therapy interventions for KOA confirm that exercise therapy and related modalities provide significant clinical benefits, with the number and frequency of sessions representing an important component of effective treatment [29]. Additionally, considering that KOA is a chronic condition without a complete cure, continuous exercise and therapy programs distributed over a larger number of sessions are frequently used in clinical practice to allow patients to achieve gradual improvement and long-term symptom reduction [30]. 

Taking all of the above into account, we consider the 20-session protocol to represent a valid and clinically relevant treatment structure.

The substantial economic burden associated with moderate to severe KOA further underscores the importance of effective interventions aimed at improving disease management and patient outcomes while reducing societal costs. Early identification and management of symptomatic KOA are essential to reducing disease burden [31,32]. KOA is increasingly recognized as a whole-joint disease amenable to prevention and early intervention, with modifiable risk factors such as obesity, joint injury, and impaired muscle function offering targets for primary and secondary prevention strategies [33,34].

Transfer learning facilitated the construction of a generalized model for predicting KOA with consistent performance across diverse cohorts. Adapting the model to population-specific risk patterns further enhances its generalizability and mitigates bias associated with ethnic or demographic imbalances in the training datasets [35].

Major advances have been made in comprehending the global burden and consequences of KOA, elucidating disease pathogenesis, and identifying individuals at risk of progression. Moving forward, progress depends on implementing public health strategies for KOA prevention, curbing the overuse of inappropriate or low-value care, promoting equitable access to cost-effective interventions, and discovering safe, inexpensive methods for pain management. Forward-looking approaches will be essential to enhance prevention, treatment, and policy for KOA [36].

Limitations

This study has several limitations. First, the relatively small sample size limits generalizability and reduces statistical power for subgroup analyses, particularly sex-specific comparisons. Second, one of the main limitations of this study is the absence of a “pure” control group receiving only kinesiotherapy. Therefore, it is not possible to completely separate the effects of the applied modalities (FMS and IFC) from their potential synergistic effect with kinesiotherapy. This factor should be taken into account when interpreting the results and is considered important for the design of future, larger-scale studies. Third, pain intensity was assessed using a single patient-reported outcome measure (VAS), which does not capture functional outcomes or long-term effects. In this study, the primary aim was to assess pain reduction, and the VAS is a widely accepted, commonly used, and highly sensitive measure. While we recognize that functional measures could provide additional information, the VAS alone is appropriate to evaluate the main outcome [15]. Additionally, blinding of participants and therapists was not feasible due to the nature of the interventions, introducing potential expectation bias, which should be emphasized. Only short-term treatment effects were evaluated, without follow-up assessment to determine durability. Finally, potential confounding factors, such as baseline physical activity level, disease severity, and concurrent non-pharmacological interventions, were not formally controlled. Taking into account the limitations, we cannot draw definitive clinical conclusions, and our findings should therefore be considered preliminary. Nevertheless, the study is strengthened by its prospective design, standardized intervention protocols, comparable baseline characteristics between groups, and the appropriate use of non-parametric statistical methods, enhancing the validity and interpretability of the findings [37,38].

## Conclusions

FMS demonstrated greater analgesic effectiveness compared to IFC in patients with KOA. These findings suggest that FMS may be more effective in this pilot cohort, as the study design (pilot study, no blinding, small n) does not allow for definitive clinical practice recommendations. Larger, multicenter studies are needed to confirm the effectiveness of FMS in a broader clinical context.
